# The impact of hydration status and fluid distribution on pulmonary function in COPD patients

**DOI:** 10.1038/s41598-022-05192-0

**Published:** 2022-01-24

**Authors:** Arturo Orea-Tejeda, Manuel Gómez-Martínez, Dulce González-Islas, Laura Flores-Cisneros, Candace Keirns-Davis, Rocío Sánchez-Santillán, Ilse Pérez-García, Nathalie Martínez-Luna, Robinson Robles-Hernández, Carlos Sánchez-Moreno, Juan José Orozco-Gutíerrez

**Affiliations:** 1 Heart Failure and Respiratory Distress Clinic, Cardiology Service at Instituto Nacional de Enfermedades Respiratorias “Ismael Cosío Villegas”, Calzada de Tlalpan 4502 Col Sec XVI, 14080 Del Tlalpan, Mexico City, Mexico; 2 Department of Clinical Research at Instituto Nacional de Cancerología, Mexico City, Mexico

**Keywords:** Medical research, Risk factors, Signs and symptoms

## Abstract

Chronic Obstructive Pulmonary Disease (COPD) patients have alterations in body composition. Bioelectrical impedance analysis (BIA) evaluates body composition, hydration status, and fluid distribution. Subjects with fluid disturbances have been found to have lower FEV_1_, respiratory muscle strength, and poor prognosis. We aimed to evaluate the effect of hydration status and fluid distribution on pulmonary function in COPD patients. A cross-sectional study, 180 patients with a confirmed diagnosis of COPD were included. Patients with asthma, advanced renal or liver disease, acute HF, exacerbation of COPD, or pacemakers were excluded. Hydration status variables (TBW, ECW, ICW) and disturbance of fluid distribution [impedance ratio (IR) > 0.84 and phase angle (PhA)] were evaluated by BIA. Pulmonary function was assessed by spirometry. The mean population age was 71.55 ± 8.94 years; 55% were men. Subjects were divided into two groups according to the IR ≥ 0.84 or < 0.84. The group with higher IR ≥ 0.84 had lower FEV_1_, FVC, FEV_1_/FVC, DLCO and, PhA compared to those with IR < 0.84. After adjusting for confounding variables TBW, ECW, IR ≥ 0.84, PhA, and resistance/height increase were associated with decreased FEV_1_. In the same way, with IR ≥ 0.84, edema index ≥ 0.48, trunk and abdominal IR were negatively associated with FVC, and PhA had a positive association with FVC. Fluid distribution, especially IR and PhA, could be a useful parameter for predicting pulmonary function in COPD patients.

## Introduction

Chronic Obstructive Pulmonary Disease (COPD) is a common, preventable, and treatable disease characterized by airflow limitation due to airway and/or alveolar abnormalities, usually caused by significant exposure to noxious particles or gases^1^, COPD is associated with several comorbidities such as hypertension, coronary artery disease, chronic renal failure, lung cancer, and complications like heart failure and cachexia affect its prognosis^2^. COPD has been considered a world health problem because it affects 600 million persons globally^3^ and is the 4th cause of global death; also, its mortality reached 30–48% in 4–7 years in persons 65–70 years old^4^.

Bioelectrical impedance analysis (BIA) is easy-to-use, noninvasive, and safe method for evaluation of body composition widely used in COPD patients^5–10^, in particular, the multifrequency modality (MF-BIA) allows evaluation of hydration status and fluid distribution. MF-BIA is considered more precise and accurate for the measurement of extracellular (ECW) and intracellular water (ICW) than single-frequency BIA^11^. Edema is not usually detectable until the interstitial fluid volume rises about 30% above average (4–5 kg of body weight). In contrast, the BIA can detect changes in tissue hydration status below 500 ml^12^.

The impedance ratio (IR) evaluates the fluid distribution between ICW and ECW and membrane integrity. IR is obtained from the ratio of impedance (Z) at a higher frequency and Z at a lower frequency (200 kHz/Z at 5 kHz/Z)^13^. Low frequencies do not pass through the cell membrane and are conducted only through ECW, while high frequencies penetrate cell membranes and are used to estimate total body water (TBW).

In COPD patients, Blasio et al. showed that patients with 5/250 kHz IR below the median had lower FEV_1_, vital capacity, respiratory muscle strength, both maximum inspiratory pressure and maximum expiratory pressure, fat-free mass index, and handgrip strength^7^. Moreover, COPD patients with sarcopenia had increased ECW/ICW ratio evaluated by 250/5 kHz IR, and this increase was even higher in severe sarcopenia ^8^. IR is a strong and independent predictor of 2-year mortality (HR: 1.16, CI 95%; 1.03–1.30) in COPD patients^10^. Likewise, the ECW/ICW ratio indicates the cellular hydration state. In COPD patients, the ECW/ICW ratio was inversely associated with Peak VO_2_^14^.

Another important marker of hydric status is the phase angle (PhA), which provides information about water distribution between ICW and ECW spaces, cellular mass, cellular integrity, and prognosis^6,8,10^.

In hemodialysis patients with end-stage renal disease, fluid overload, assessed by MF-BIA, is associated with restrictive and obstructive respiratory abnormalities^15^. However, few studies have evaluated the hydric alterations in COPD patients^7,8,10^ and its impact on pulmonary function. This study aimed to evaluate the impact of hydration status and fluid distribution on pulmonary function in COPD patients.

## Methods

### Study design

A cross-sectional study was carried out in the Cardiology Service at Instituto Nacional de Enfermedades Respiratorias “Ismael Cosío Villegas” from August 1, 2019, to March 31, 2020.

Patients with a confirmed diagnosis of COPD according to the Global Initiative for Chronic Obstructive Lung Disease (GOLD) recommendations^16^ were included. Age > 40 years old, smoking record (tobacco index > 10), or wood smoke or biomass exposure (> 200 h/year), and spirometry with a post-bronchodilator FEV_1_/FVC ratio < 0.70^1^ constituted inclusion criteria. Patients with asthma, advanced renal or liver disease, acute HF, exacerbation of COPD, pacemakers, acute coronary syndromes, percutaneous transluminal coronary angioplasty, or coronary artery bypass graft within the prior three months were excluded.

The study was conducted according to the Declaration of Helsinki and was approved by the Institutional Ethics and Research Committee of Biomedical Research in Humans of the Instituto Nacional de Enfermedades Respiratorias “Ismael Cosío Villegas” (approval number E-02-18).

### Outcome measures

Body composition, anthropometry, pulmonary function, clinical and demographic variables, which are part of the clinical management of the patients who come to our Institute, were evaluated.

### Anthropometry

Weight and height were measured according to the manual reference of anthropometric standardization^17^; all subjects wore light clothing and were barefoot. Body mass index was calculated by dividing the total body weight (kilograms) by the squared height (meters).

### Bioelectrical impedance analysis (BIA)

Total body composition and raw variables were measured with whole-body bioelectrical impedance analysis using four-pole multifrequency equipment BodyStat QuadScan 4000 (BodyStat, Isle of Man, UK) by standard technique^18^: The measurements were conducted by the same operator, in the morning, in a comfortable area, free of drafts, with portable electric heaters. The area was cleaned before the study. The subjects were fasting and should not have exercised eight hours before or consumed alcohol 12 h before the study. During the entire study, the person was in a supine position with the arms separated from trunk by about 30° and the legs separated by about 45°. Electrodes were placed on the hand and ipsilateral foot. We registered the resistance, reactance, PhA, and IR.

Impedance ratio (IR) was calculated as follows: the ratio of high (200 kHz) to low frequency (5 kHz) of multifrequency BIA. In our population, the 50-percentile value was 0.84.

#### Segmental impedance ratio

The segmental impedance ratio was performed in the same position as whole-body bioimpedance. Segmental IR was calculated as the ratio of high (200 kHz) to low frequency (5 kHz).

Trunk impedance ratio: A pair of electrodes was placed on the midline at the interclavicular level, and the second pair was placed on the right upper ventral iliac spine.

Thorax impedance ratio: A pair of electrodes was placed on the midline at the interclavicular level, second pair placed on the right costal border.

Abdomen impedance ratio: A pair of electrodes was placed on the midline at the xiphoid process level, a second pair placed on the right upper ventral iliac spine.

Edema index was calculated as follows: the ratio between ECW (lt) to TBW (lt). In our population, the 75-percentile value was 0.48.

### Handgrip strength

Handgrip strength was measured with a mechanical Smedley Hand Dynamometer (Stoelting, Wood Dale, UK) according to the technique described in Rodriguez et al.^19^.

### Pulmonary function

Spirometry testing was conducted by an experienced respiratory medicine technician using a portable spirometer (EasyOnePC, Ndd Medical Technologies Inc., Zürich, Switzerland) according to the standards of the American Thoracic Society/European Respiratory Society^20^. The spirometry variables analyzed were the Forced Expiratory Volume in the first second (FEV_1_) and the Forced Vital Capacity (FVC) after administration of a bronchodilator. After 15 min at rest, the participant performed a maximum forced inhalation and a powerful forced expiration using a nose clip. The reference values for spirometry were obtained for Mexican–American individuals^21^.

### Statistical analysis

Analyses were performed using a commercially available STATA version 14 (Stata Corp., College Station, TX, U.S.A.). Categorical variables were presented as frequencies and percentages; continuous variables with normal distribution were presented as mean and standard deviation. The Shapiro–Wilk test was used to test the normality of continuous variables. Normal continuous variables were presented as mean and standard deviation, while non-normal variables were presented as median and percentiles 25–75. A comparison among study groups was analyzed with X^2^ for categorical variables and unpaired Student's t-test or Mann‐Whitney U tests for continuous variables.

Linear regression analysis was performed to examine the association between pulmonary function (FEV_1_% predicted and FVC % predicted) and hydration status and fluid distribution. The models were adjusted for sex, height, and age. A *p* < 0.05 was considered statistically significant.

### Ethics approval and consent to participate

This study was conducted in accordance with the Declaration of Helsinki and was approved by the Institutional Ethics, and Research Committee of Biomedical Research in Humans of the Instituto Nacional de Enfermedades Respiratorias “Ismael Cosío Villegas” and all participants gave informed consent.

## Results

One hundred and eighty patients with stable COPD were included. The mean population age was 71.55 ± 8.94 years; 55% were men. Subjects were divided in two groups according the IR ≥ 0.84 (n = 88) or < 0.84 (n = 92). Table 1 shows the basal characteristics of the patients according to IR. The group with higher IR ≥ 0.84 had fewer men (44.32% vs 65.22%, *p* = 0.005), were older, (74.27 ± 7.85 vs 68.95 ± 9.19, *p* < 0.001) and had lower albumin levels (3.33 ± 0.65 mg/dl vs 4.07 ± 0.49 mg/d, *p* = 0.011). FEV_1_ was lower (lts: 1.01 ± 0.44 vs 1.40 ± 0.66, *p* < 0.001; %: 50.35 ± 21.50 vs 58.87 ± 21.83, *p* = 0.009) as was FVC (lts: 2.04 ± 0.66 vs 2.55 ± 0.91, *p* < 0.001; %: 71.06 ± 18.65 vs 78.36 ± 18.52, *p* = 0.009). The FEV_1_/FVC was lower (0.50 ± 0.13 vs 0.54 ± 0.13, *p* = 0.041) and the carbon monoxide lung diffusion capacity (DLCO) as well (52.07 ± 28.77% vs. 76.51 ± 31.06%, *p* = 0.014). The same was true of weight (64.23 ± 15.31 kg vs 69.65 ± 16.08 kg, *p* = 0.021) handgrip strength (20.76 ± 7.41 kg vs 26.06 ± 8.25 kg, *p* < 0.001), skeletal muscle mass index (7.61 ± 1.65 kg/m vs 8.77 ± 1.79, *p* < 0.001) and PhA (4.46 ± 0.47 vs 5.79 ± 0.92, *p* < 0.001) compared to those with IR < 0.84. The left atrial volume index was higher (37.6 ± 11.98 vs. 33.51 ± 8.16, *p* = 0.078). Table 2 shows the association between hydration status and fluid distribution with FEV1 and FVC adjusted by age, sex, and height. TBW, ECW, IR ≥ 0.84, and resistance/height increase were associated with FEV1 decrease, while PhA was associated with FEV1 increase. Likewise, with the IR ≥ 0.84 and the edema index ≥ 0.48, trunk and abdominal IR were negatively associated with FVC, while the PhA had a positive association with FVC.

**Table 1:** Clinical characteristics according to impedance ratio.

	All n = 180	Impedance ratio ≥ 0.84 n = 88	Impedance ratio < 0.84 n = 92	*p*-value
**Demographic characteristics**				
Male, n (%)	99 (55)	39 (44.32)	60 (65.22)	0.005
Age, years	71.55 ± 8.94	74.27 ± 7.85	68.95 ± 9.19	< 0.001
Albumin, mg/dl	3.80 ± 0.65	3.33 ± 0.65	4.07 ± 0.49	0.011
Tobacco use, n (%)	130 (72.63)	65 (73.86)	65 (71.86)	0.715
Tobacco index,pack-yr	40 [20–60]	36 [15 to 51.75]	40 [20 to 60]	0.453
Biomass, n (%)	73 (41.24)	40 (45.98)	33 (36.67)	0.208
Biomass index, h/yr	180 [50–360]	219 [75 to 390]	128 [50 to 360]	0.140
**GOLD classification**				
1-2	99 (55)	42 (47.73)	57 (61.96)	0.055
3-4	81 (45)	46 (52.27)	35 (38.04)	
**Comorbidities**				
Diabetes, n (%)	41 (22.78)	18 (20.45)	23 (25)	0.467
Hypertension, n (%)	88 (48.89)	49 (55.68)	39 (42.39)	0.075
Obesity, n (%)	45 (25)	17 (19.32)	28 (30.43)	0.085
Heart failure, n (%)	67 (37.22)	32 (36.36)	35 (38.04)	0.816
Right heart failure, n (%)	42 (23.33)	16 (18.18)	26 (28.26)	0.110
**Echocardiography data**				
Left atrial volume index ml/m^2^	35.41 ± 10.23	37.67 ± 11.98	33.51 ± 8.16	0.078
Right atrial volume index ml/m^2^	32.62 ± 12.24	34.05 ± 10.84	31.49 ± 13.27	0.360
PSAP, mmHg	43.04 ± 16.87	43.45 ± 18.60	42.63 ± 15.13	0.809
**Pulmonary function**				
FEV_1_, % predicted	54.71 ± 22.03	50.35 ± 21.50	58.87 ± 21.83	0.009
FEV_1_, lt	1.21 ± 0.59	1.01 ± 0.44	1.40 ± 0.66	< 0.001
FVC, % predicted	74.79 ± 18.89	71.06 ± 18.65	78.36 ± 18.52	0.009
FVC, lt	2.30 ± 0.83	2.04 ± 0.66	2.55 ± 0.91	< 0.001
FEV_1_/FVC	0.52 ± 0.13	0.50 ± 0.13	0.54 ± 0.13	0.041
DLCO, %	63.68 ± 31.98	52.07 ± 28.77	76.51 ± 31.06	0.014
pH	7.41 ± 0.04	7.40 ± 0.05	7.43 ± 0.03	0.384
PaO2, mmHg	54.95 [47.95− 69.6]	59.3 [49.2 to 63.4]	54 [49.2 to 73]	0.984
PaC02, mmHg	37.9 [33.3 to 44.8]	43 [36.7 to 47.4]	35.2 [34 to 41.5]	0.096
HCO3, meq/L	22.7 [20.6 to 25.9]	24.7 [20.8 to 32.2]	23.5 [21 to 25.3]	0.296
Saturation, %	86.87 ± 8.35	87.55 ± 7.86	86.14 ± 9.11	0.671
FiO_2_, %	21 [21–21]	21 [21–30]	21 [21–25]	0.691
Lactate, mmol/lt	1.22 ± 0.54	1.25 ± 0.64	1.18 ± 0.40	0.762
Residual volume, lt	3.93 ± 1.62	4.13 ± 2.03	3.81 ± 1.37	0.214
Total lung capacity, lt	6.84 ± 1.79	7.04 ± 1.27	6.65 ± 2.2	0.144
**Blood electrolyte**				
Sodium, mmol/	138.56 ± 3.53	138.32 + 4.19	138.81 ± 2.82	0.433
Potassium, mmol/L	4.48 ± .55	4.59 ± 0.54	4.36 ± 0.56	0.324
Chlorine, mmol/L	102.86 ± 3.48	101.75 ± 2.70	104.09 ± 3.93	< 0.001
**Body composition**				
Weight, kg	67.00 ± 15.90	64.23 ± 15.31	69.65 ± 16.08	0.021
Height, cm	157.78 ± 11.36	156.70 ± 11.87	158.80 ± 10.81	0.215
Body mass index, kg/m^2^	26.86 ± 5.39	26.17 ± 5.43	27.52 ± 5.29	0.092
Handgrip strength, kg	23.37 ± 8.25	20.76 ± 7.41	26.06 ± 8.25	< 0.001
Skeletal muscle mass index, kg/m	8.20 ± 1.81	7.61 ± 1.65	8.77 ± 1.79	< 0.001
Total body water, %	52.97 ± 8.78	52.88 ± 9.07	53.05 ± 8.54	0.893
Extracellular water, %	24.94 ± 6.67	25.10 ± 6.57	24.79 ± 6.79	0.758
Intracellular water, %	28.67 ± 6.16	27.76 ± 4.95	29.39 ± 6.93	0.189
Edema index	0.48 ± 0.15	0.48 ± 0.11	0.49 ± 0.18	0.845
Third space, lt	0.1 [− 0.4 to 0.8]	0.2 [− 0.25 to 0.9]	0 [− 0.5 to 0.7]	0.070
Phase angle, º	5.14 ± 0.99	4.46 ± 0.47	5.79 ± 0.92	< 0.001

*GOLD classification* Global Initiative for Chronic Obstructive Lung Disease classification, *FEV*_*1*_ Forced expiratory volume in 1 s, Forced vital capacity, *DLCO* Carbon monoxide diffusing capacity, *PaO*_*2*_ Partial pressure of oxygen, *PaCO*_*2*_ Partial pressure of carbon dioxide, *HCO*_*3*_ Bicarbonate, *FiO*_*2*_ Fraction of inspiratory oxygen.

**Table 2:** Impact of hydration status and fluid distribution over pulmonary function**.**

	FEV 1			FVC		
	β	IC 95%	*p*	β	IC 95%	*p*
Total body water, %	− 0.404	− 0.80 to − 0.006	0.046	0.098	− 0.253 to 0.450	0.581
Extracellular Water,%	− 0.508	− 0.960 to − 0.056	0.028	− 0.090	− 0.491 to 0.310	0.657
Intracellular Water %	0.162	− 0.580 to 0.904	0.666	0.443	− 0.188 to 1.075	0.166
Third Space Water, lt	− 2.811	− 5.864 to 0.241	0.071	− 0.556	− 3.246 to 2.132	0.684
IR ≥ 0.84	− 13.149	− 19.671 to − 6.627	< 0.001	− 9.260	− 15.092 to − 3.429	0.002
Edema index ≥ 0.48	− 3.76	− 12.05 to 4.52	0.371	− 9.75	− 16.88 to− 2.62	0.008
Phase angle,º	7.76	4.179 to 11.348	< 0.001	3.254	0.555 to 5.954	0.018
Resistence/height	− 0.06	− 0.11 to − 0.01	0.015	− 0.02	− 0.07 to 0.02	0.323
Reactance/height	0.19	− 0.23 to 0.62	0.373	0.31	− 0.06 to 0.70	0.104
**Segmentary IR**						
Trunk IR ≥ 0.84	− 0.375	− 10.992 to 10.24	0.944	− 8.899	− 17.588 to 0.209	0.045
Thorax IR ≥ 0.84	0.071	− 10.650 to 10.794	0.989	− 3.057	− 11.874 to 5.759	0.492
Abdomen IR ≥ 0.84	− 3.120	− 19.015 to12.77	0.695	− 13.928	− 27.743 to − 0.113	0.048

*IR* Impedance ratio. Variables were adjusted by sex, height, and age.

## Discussion

The main finding in our study was the significance of the impact of hydration status and fluid distribution on pulmonary function in COPD patients.

Several studies have found that weight increase is frequently associated with functional class decreases and pulmonary congestion in the absence of clinical edema^24,25^, although the pathophysiology of impaired pulmonary function has not been completely elucidated. In some cases, respiratory symptoms are either underestimated or overlooked in clinical practice^22^. However, the methods for estimating body composition like imaging technique and isotope dilution to evaluate hydration status are relatively invasive, expensive, and not suitable in clinical practice^12,23^. The BIA is a non-invasive, inexpensive, and easily reproducible procedure that detects fluid overload and fluid distribution^24^. This method has already been validated in several pathologies, demonstrating that an IR higher than 0.80 is associated with fluid overload^25^ and is an independent predictor of mortality in hemodialysis and COPD patients^7,26^.

Lower values of IR and higher values of PhA provide information about the fluid distribution between ICW and ECW compartments. Moreover, they are considered indicators of greater cellularity, cell membrane integrity, and nutritional status. IR disturbance is associated with diminished pulmonary function, lower peripheral and respiratory muscle strength, fat-free mass index, physical function, and poor prognosis in COPD patients^7,10^. In this study, we observed that IR ≥ 0.84 was an independent predictor for FEV_1_ (β: − 13.149, CI 95%; − 19.671 to -6.627, *p* < 0.001) and FVC (β: − 9.260, CI 95%; − 15.092 to − 3.429, *p* < 0.002) adjusted for age, sex and height. The subjects with IR ≥ 0.84 had a 13.14% lower FEV_1_ and 9.26% lower FVC than subjects with IR < 0.84 in this population.

In COPD patients, low PhA was associated with low FEV_1_, low skeletal muscle mass, diminished physical function, increased disease severity, malnutrition, poor quality of life, longer hospital stay, exacerbations, and poor prognosis^6,10^. Our results showed that the PhA is a strong independent predictor of FEV_1_ (β: 7.76, CI 95%; 4.179 to 11.348, *p* < 0.001) and FVC (β: 3.25, CI 95%; 1.077–6.665, *p* = 0.018) adjusted by age, sex, and height. That is, for every 1 degree that PhA increases, there is an increment of 7.76% for FEV_1_ and 3.25% in FVC in this population.

Our study also showed a negative association between FEV1, with TBW, ECW, IR > 0.84, and resistance adjusted by height. And a negative association of FVC with edema index > 0.47. Similar results have been observed in different studies Lui et al., found that the edema index evaluated by ECW/TBW was > 0.39 by multifrequency BIA in acute heart failure patients (20–80 years old) hospitalized in cardiogenic shock. These patients had an elevated risk of re-hospitalization related to HF (OR: 4.14, IC 95%; 1.05–15.28, *p* = 0.04)^27^. Also, Androne et al., found that blood volume increase was associated with higher pulmonary wedge pressure and mortality risk in heart failure patients with no visible edema (39% in a year)^28^. These data suggest that the mechanism of fluid overload may relate to redistribution rather than volume overload^29,30^.

Fluid overload, together with a potential increase in pulmonary capillary permeability, can result in pulmonary edema and pleural effusion, abnormalities that could at least in part explain the reported decrease in pulmonary function in end-stage renal disease patients^31^. In these cases, hemodialysis has been able to remove the excess fluid body. As other studies have demonstrated^15^, this improves pulmonary function with improved spirometric parameters before and after hemodialysis sessions associated with weight reduction^32^. In addition, fluid overload was also observed with significant weight increases in the periods between dialysis accompanied by declines in pulmonary function, which again improved with hemodialysis. Our study showed a similar result; trunk and abdominal IR ≥ 0.84 were inversely related to FVC.

Similarly, a congestive state is a common complication in heart failure patients, depending on the type of heart failure type. If the left ventricle is affected it is in the thorax, and abdominal when the right ventricle is responsible for hemodynamic alterations. In those patients with reduced ejection fraction or in most of the patients with concomitant COPD with preserved ejection fraction, left ventricular filling pressure is accompanied by elevated wedge pulmonary pressure with resulting engorgement of the pulmonary vasculature, bronchial vasculature, and capillaries, and fluid retention into the lung interstitial space. The outcome is “both restrictive—reductions in FVC and total lung capacity—and obstructive—reductions in peak expiratory flow and maximal mid expiratory flow (FEF25-75)”^33^. In experimental models with saline infusion, Brown et al. observed a reduction of airway area, suggesting that a potential increase in pulmonary capillary permeability and edema in the small bronchioles could lead to structural changes in the walls and increased airway resistance.

On the other hand, with exercise chronic heart failure patients increase their ventricular filling pressures and decompensation periods due to volume overload or redistribution, raise pulmonary capillary wedge pressure, and allow fluid transudation into the interstitial space with the formation of edema^34^.

In COPD patients, heart failure is not always recognized, even though up to 23% of them are hospitalized^35^, and 50% die from cardiovascular causes, more than from respiratory distress^36^. Moreover, pulmonary congestion during COPD exacerbations increases the risk of dying, possibly due to heart failure^37^. The interstitial fluid or intravascular volumes depend on many factors, including HF etiology^38,39^. One of them, hypoalbuminemia, is partly responsible for the peripheral congestive state common in heart failure^40,41^ when capillary membrane stabilization capacity through colloid osmotic properties is severely compromised^42^.

dditionally, there is an interaction between albumin levels with the hydrostatic pressure factors and the fluid redistribution that affect lung function and pulmonary gas exchange independent of heart failure phenotypes^42,43^. Accumulation of pulmonary blood volume has been associated, as in our cases, with reduced spirometric parameters and a reduced DLCO. This increase of extravascular lung water is the main pathogenic factor of diminished functional capacity^44^. Our findings correlate with these pathophysiological mechanisms; the IR ≥ 0.84 with a significant FEV_1_ reduction was similar for FVC. Moreover, in patients with an IR ≥ 0.84, the DLCO was significantly reduced compared to decreased skeletal muscle mass index, handgrip strength, albumin, and worse functional spirometric parameters. However, when the left atrial volume index was higher, meaning left ventricular filling pressures were increased, a pathophysiological scenario of pulmonary fluid overload or local redistribution would be the outcome. The decrease in DLCO levels can be multifactorial and can lead to a greater number of exacerbations and a reduction in the quality of life as well as an increase in dyspnea.

### Limitations and strengths

Our first limitation is the small sample size and the fact that it is a cross-sectional study. On the other hand, important previous studies have observed that subjects with hydration status and fluid distribution disturbances assessed by BIA have a worse pulmonary function. However, these studies performance a bivariate analysis that does not evaluate whether there is an independent association between the study variables. Among the strengths of our study, this is the first study in COPD patients that evaluates the association hydration status and fluid distribution on pulmonary function performance a multivariate prediction model adjusted for confounding variables in COPD patients, which allows us to assess the effect size of water status variables on FEV1 and FVC.

## Conclusions

Hydration status and fluid distribution have a significant impact on pulmonary function. Early detection of these disturbances in BIA, especially by IR and PhA, enables the early application of therapeutic strategies to improve pulmonary function in COPD patients.

## Data Availability

Data are available upon reasonable request.

## Abbreviations

BIA: Bioelectrical impedance analysis
COPD: Chronic Obstructive Pulmonary Disease
ECW: Extracellular water
ICW: Intracellular water
IR: Impedance ratio
MF-BIA: Multifrequency bioelectrical impedance analysis
PhA: Phase angle
TBW: Total body water
